# The ceRNA Network Has Potential Prognostic Value in Clear Cell Renal Cell Carcinoma: A Study Based on TCGA Database

**DOI:** 10.1155/2020/4830847

**Published:** 2020-06-26

**Authors:** Haosheng Liu, Zhaowen Zhu, Jianxiong Fang, Tianqi Liu, Zhenhui Zhang, Chao Zhao, Xiaoyong Pu, Jiumin Liu

**Affiliations:** ^1^Guangdong Provincial People's Hospital, Guangdong Academy of Medical Sciences, School of Medicine, South China University of Technology, Guangdong Guangzhou, China; ^2^Department of Breast Surgery, Breast Tumor Center, Sun Yat-sen Memorial Hospital, Sun Yat-sen University, Guangdong Guangzhou, China; ^3^Guangdong Provincial People's Hospital, Guangdong Academy of Medical Sciences; The Second School of Clinical Medicine, Southern Medical University, Guangdong Guangzhou, China; ^4^Shantou University Medical College, Guangdong Provincial People's Hospital, Guangdong Academy of Medical Sciences, Guangdong Guangzhou, China; ^5^Department of Urology, Guangdong Provincial People's Hospital, Guangdong Academy of Medical Sciences, Guangdong Guangzhou, China

## Abstract

Clear cell renal cell carcinoma (ccRCC) is a very common cancer in urology. Many evidences suggest that complex changed pathways take a nonnegligible part in the occurrence and development of ccRCC. Nevertheless, the underlying mechanism is not clear. In this study, the expression data between ccRCC and normal tissue samples in TCGA database were compared to distinguish differentially expressed genes (DEGs: mRNAs, miRNAs, and lncRNAs). Afterwards, we used GO enrichment and KEGG pathway enrichment analyses to explore the functions of these DEGs. We also found the correlation between three RNAs and created a competing endogenous RNA (ceRNA) network. Moreover, we used univariate Cox regression analysis to select DEGs that are connected with overall survival (OS) of ccRCC patients. We found 1652 mRNAs, 1534 lncRNAs, and 173 miRNAs that were distinguished in ccRCC compared with normal tissues. According to GO analysis, the maladjusted mRNAs are mainly concentrated in immune cell activation and kidney development, while according to KEGG, they are mainly concentrated in pathways related to cancer. A total of 5 mRNAs, 1 miRNA, and 4 lncRNAs were connected with patients' OS. In this article, a network of lncRNA-miRNA-mRNA was established; it is expected to be able to indicate possible molecular mechanisms for initial of ccRCC and provide a new viewpoint for diagnosis of ccRCC.

## 1. Introduction

RCC accounts for about 3% of human cancers; in the last 20 years, morbidity has increased about 2% per year both worldwide and in Europe, causing about 99200 RCC patients and 39100 RCC-related deaths in EU in 2018 [1]. RCC is the commonest kidney essence lesion, accounting for approximately 90% of all renal malignances. Many patients with renal mass have no symptoms until the terminal stage. At present, >60% of RCC patients are discovered by ultrasound (US) or CT performed for other disease. Flank pain, visible haematuria, and palpable abdominal mass which are classical signs of RCC are less now [2]. Therefore, an in-depth study of the biomarkers of tumorigenesis and progression in RCC is badly needed to gain the solutions and realize diagnosis goals.

In the human genome, protein-coding genes account for less than 3 percent; however, more than 80% of our genomes have no protein-coding capacity. Such transcription is called noncoding RNAs (ncRNAs), which are divided into long noncoding RNAs (lncRNAs) and small noncoding RNAs (sncRNAs). More and more evidences showed that lncRNAs are connected with tumorigenesis and development through a variety of biological processes, including transcriptional regulation, self-renewal, and carcinogenesis [3]. The rapid development of RNA-Seq technology allows people to discover new lncRNAs related to urologic malignancies [4]. The article analyzed the differentially expressed lncRNAs and their function in ccRCC.

microRNAs are sncRNAs with regulatory function [5]. After binding to the 3′-UTR of mRNA, it can inhibit the process of transcription and even induce mRNA degradation. However, miRNAs are also influenced by other ncRNAs, for example, lncRNAs and circular RNAs (circRNAs), which serve as sponges for miRNAs [6, 7]. Currently, dysregulated miRNAs are believed to be connected with the occurrence of tumors including gastrointestinal cancer [8], colon cancer [9], prostate cancer [10], and colorectal cancer [11]. Thus, miRNAs may be a good biomarker for cancer diagnosis.

Competitive endogenous RNA theory indicated that RNAs which have miRNA binding sites struggle for posttranscriptional control. This theory has got wide interest as a whole function of lncRNAs and circRNAs and a substitution function of mRNAs. Moreover, the hypothesis assumes that specific RNAs can regulate the expression of miRNA target gene by inhibiting miRNA activity [12]. However, there are few studies on ceRNA of ccRCC.

We explored the interaction among three differentially expressed RNAs from The Cancer Genome Atlas (TCGA) database. In addition, these RNAs were annotated for possible biological functions. Then, we established a ceRNA network in ccRCC. Besides, we used K-M and receiver operating characteristic analysis to distinguish prognostic DEGs for predicting OS of ccRCC. Our results provide new insights into diagnosis strategies for ccRCC.

## 2. Materials and Methods

### 2.1. Data Download and Processing

We downloaded ccRCC patients' transcriptome profiling and clinical data from TCGA database (https://tcga-data.nci.nih.gov/tcga/), which was inputted on the Illumina HiSeq RNA-Seq platform. The exclusion criteria were (i) samples without clinical data and (ii) samples without complete information of the stage and overall survival period. Finally, a total of 534 cases contain 472 clear cell renal cell carcinoma tissues and 62 adjacent nontumor renal tissues were included in our study. We annotated the names of RNAs through Homo sapiens.GRCH38.84.chr.gtf.gz downloaded from Ensembl Genome Browser 97 (http://www.ensembl.org). Both RNA profile data and clinical characteristics of ccRCC are publicly available and in open-access platforms. Therefore, approval by local ethics committee was not needed.

### 2.2. Analysis of Differentially Expressed Genes

We used the edgeR package to make comparisons between tumor tissues and normal specimens to select the DEGs. DEmRNAs and DElncRNAs were distinguished through the threshold of a log fold change (logFC) > 2 and an adj. *P* value < 0.05, and the DEmiRNAs was identified using the threshold of a logFC > 1 and an adj. *P* value < 0.05.

### 2.3. Cluster Analysis of the DEGs

R software was used for cluster analysis based on the expression value of each sample. With R package “pheatmap,” hierarchical clustering analysis was conducted. We showed the results through a clustergram. The column means the samples, and the row shows the gene expression level.

### 2.4. Functional Enrichment Analysis

We analyzed the DEGs' functional enrichment with the online bioinformatics tools KOBAS 3.0 (http://kobas.cbi.pku.edu.cn/index.php) and DAVID (https://david.ncifcrf.gov/, version 6.8). KEGG pathways and GO terms were considered to be statistically significant when *P* value < 0.05.

### 2.5. Predict the Target of miRNAs

We used miRDB (http://www.mirdb.org/), TargetScan (http://www.targetscan.org/), and miRTarBase (http://amp.pharm.mssm.edu/Harmonizome/resource/MiRTarBase) to predict the possible target genes of DEmiRNAs. The overlapped target genes were searched by Venn overlap analysis. miRNAs' target lncRNAs were predicted through the miRcode dataset (http://www.mircode.org/).

### 2.6. Construction of the ceRNA Network

We identified DEGs and the relationships between miRNA-mRNA and miRNA-lncRNA. Based upon these results, Cytoscape 3.6.1 (version 3.6.1, San Diego CA) was used to establish the ceRNA network.

### 2.7. Construction of the Protein-Protein Interaction (PPI) Network

To explore the interaction between mRNAs involved in this ceRNA network, a PPI network was constructed by the Search Tool for the Retrieval of Interacting Genes (http://string.embl.de/) with a composite score greater than 0.4 as the cut-off criterion [13].

### 2.8. Survival and Receiver Operating Characteristic Analyses

We used a univariate Cox regression model to analyze the connection between ccRCC patients' OS and ceRNA network DEGs. *P* value < 0.05 was regarded as statistically significant. Then, those DEGs were presented with the survival curve. The cut-off point divided all samples into high and low expression groups. In addition, sensitivity and specificity were calculated through ROC curves and AUC values.

### 2.9. Statistical Analysis

We analyzed the data through R and Cytoscape software. Data were expressed as mean ± standard deviation. The differences between two groups were analyzed through fold change and Student's *t*-test. The survival curves were constructed by K-M method, and log-rank tests were used to test survival differences. When *P* < 0.05, we thought the difference are significant.

## 3. Results

### 3.1. Clustering Analysis of DEGs

**Figure 1:**
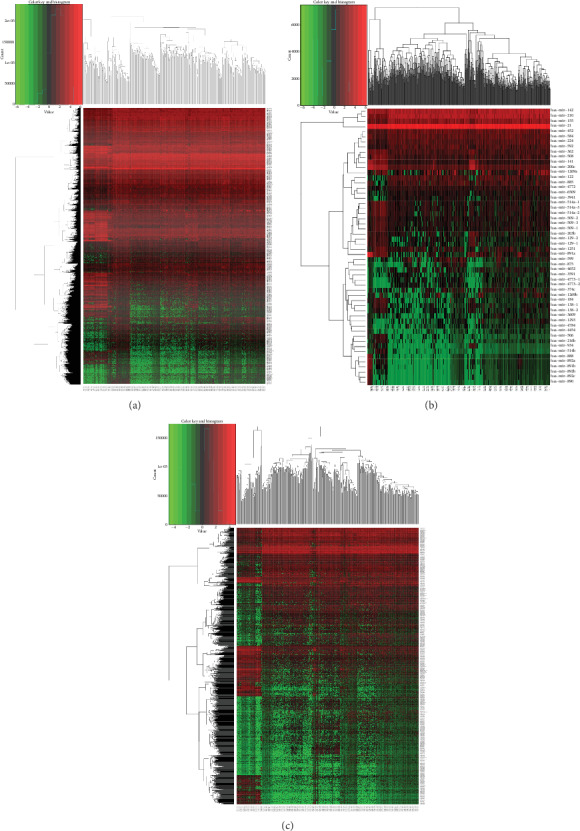
Cluster analysis of differentially expressed genes. (a) mRNAs; (b) miRNAs; (c) lncRNAs. The rows represent differentially expressed mRNAs, miRNAs, and lncRNAs, and the columns represent every sample. Red represents high expression, and green represents low expression. mRNAs: messenger RNA; miRNAs: microRNAs; lncRNAs: long noncoding RNAs.

The number of DEGs was 1652 (mRNA), 173 (miRNA), and 1534 (lncRNA), respectively. Hierarchical clustering analysis was used to analyze all the DEGs. (Figure 1). Besides, we showed the top 10 upregulated and downregulated DEGs in ccRCC (Tables 1–3).

### 3.2. GO Enrichment Analysis of DEmRNAs

**Figure 2:**
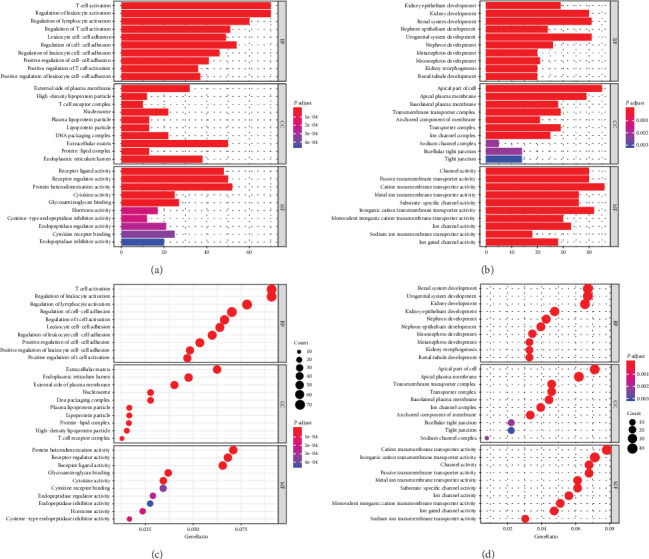
Significantly enriched Gene Ontology (GO) terms of DEmRNAs. (a, c) Upregulated genes. (b, d) Downregulated genes.

We used DAVID database to analyze these dysregulated genes and perform enrichment analysis to determine their respective functions. The upregulated genes were mainly enriched in T cell activation and regulation of leukocyte activation (Figures 2(a) and 2(c)). The downregulated genes were mostly enriched in renal system development and urogenital system development (Figures 2(b) and 2(d)).

### 3.3. KEGG Pathway Enrichment Analysis of DEmRNAs

**Figure 3:**
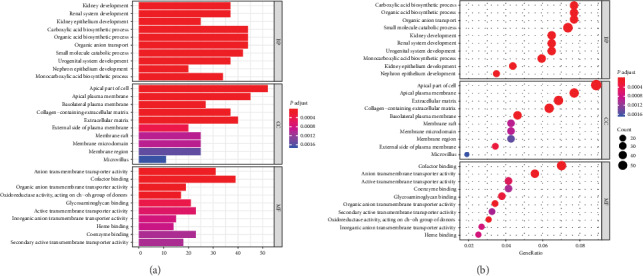
Kyoto Encyclopedia of Genes and Genomes (KEGG) pathway enrichment analysis of DEmRNAs.

The KEGG pathway enrichment method was also used to analyze the DEmRNAs. These DEmRNAs had the strongest correlation with neuroactive ligand-receptor interaction, cytokine-cytokine receptor interaction, metabolism of xenobiotics by cytochrome P450, cell adhesion molecules (CAMs), and complement and coagulation cascades (Figure 3). Based on these results, we believed that the DEmRNAs play an indispensable part in the progression of ccRCC.

### 3.4. ceRNA Network

**Figure 4:**
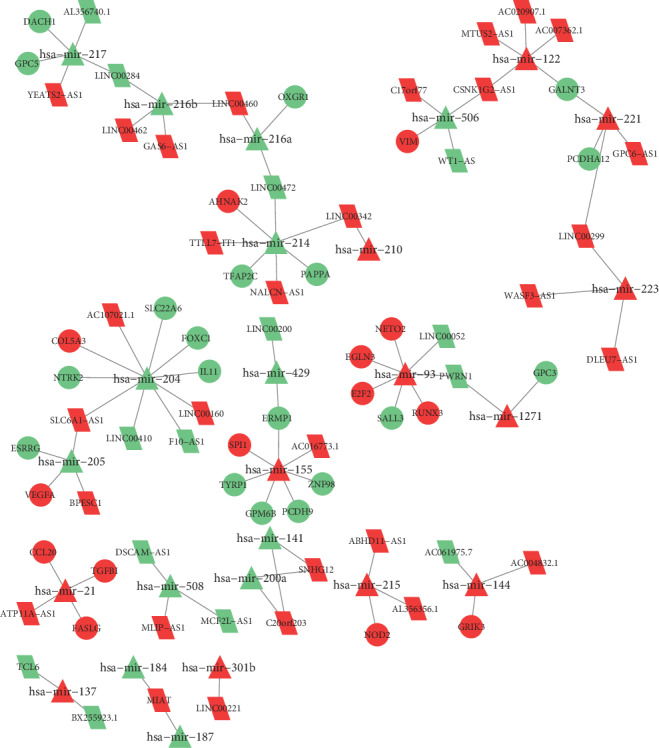
The ceRNA network. The triangles denote miRNAs, circles denote mRNAs, and rhombuses denote lncRNAs. Red and green represent upregulated and downregulated genes, respectively. The size of the point represents the expression of a given RNAs, whereas the larger points indicate a higher expression.

There were 44 DElncRNAs that may bind to 25 DEmiRNAs. We searched for the DEmiRNAs' target mRNAs through using miRDB, miRTarBase, and TargetScan. Based on the Venn intersection analysis, 33 DEmRNAs were identified. At last, we established a ceRNA network (Figure 4).

### 3.5. Protein-Protein Interaction Network

**Figure 5:**
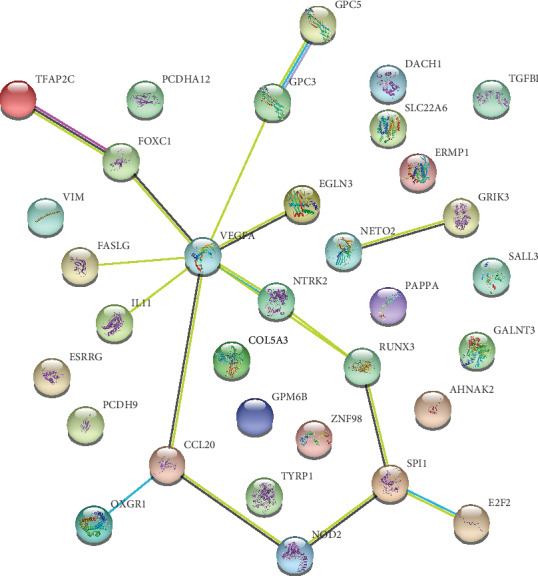
PPI network.

To clarify the relationships between the 33 DEmRNAs involved in this ceRNA network, a PPI network was constructed using the STRING database containing 32 nodes and 15 edges (Figure 5). KEGG pathway enrichment analysis of these mRNAs was performed with KOBAS 3.0 with *P* < 0.05 as the cut-off criterion. The results showed that these mRNAs were mainly enriched in pathways related to cancer (Table 4).

### 3.6. Analysis of the Survival-Associated DEGs

**Figure 6:**
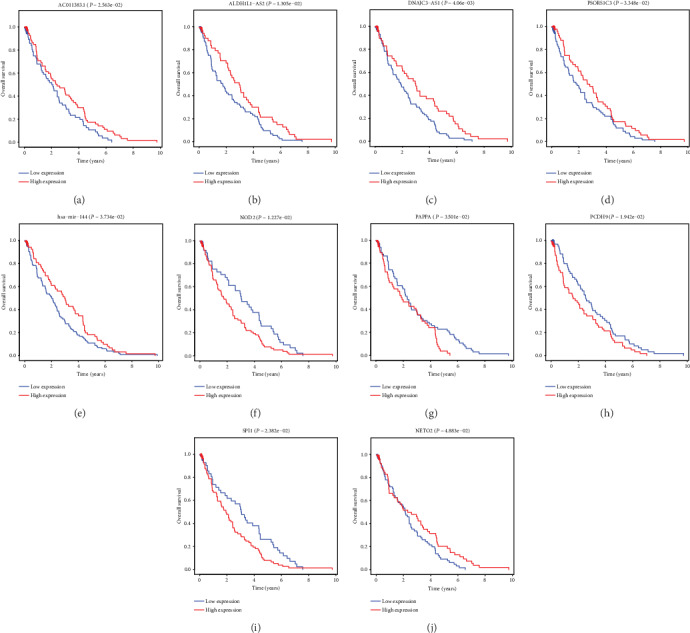
Kaplan-Meier survival curves for DEGs as independent prognostic factors associated with OS in ccRCC. ccRCC: clear cell renal cell carcinoma; DEGs: differentially expressed genes; OS: overall survival. *P* < 0.05 was considered statistically significant. (a–d) DElncRNAs; (e) DEmiRNA; (f–j) DEmRNAs.

**Figure 7:**
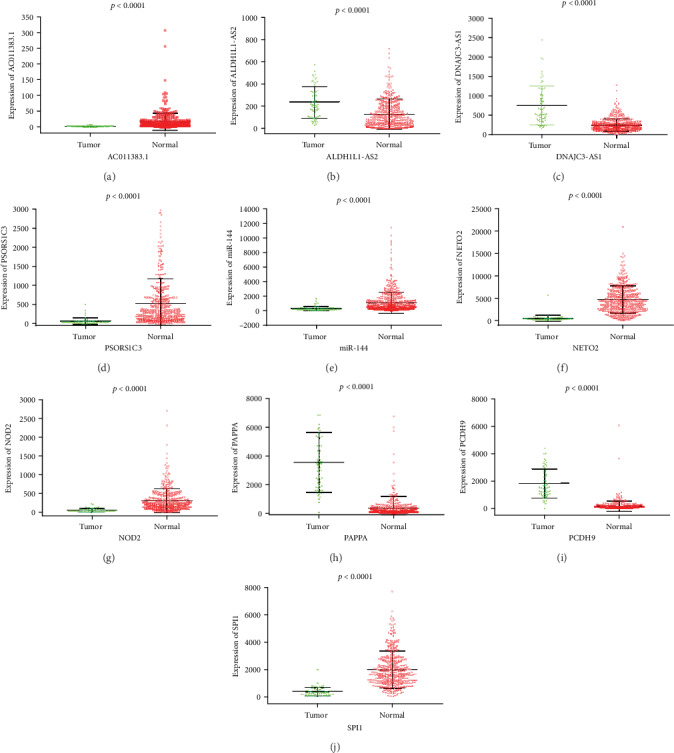
Analysis of DEG levels in human ccRCC. The green and red dots represent cancer and paracancerous tissues, respectively. (a–d) DElncRNAs; (e) DEmiRNA; (f–j) DEmRNAs.

**Figure 8:**
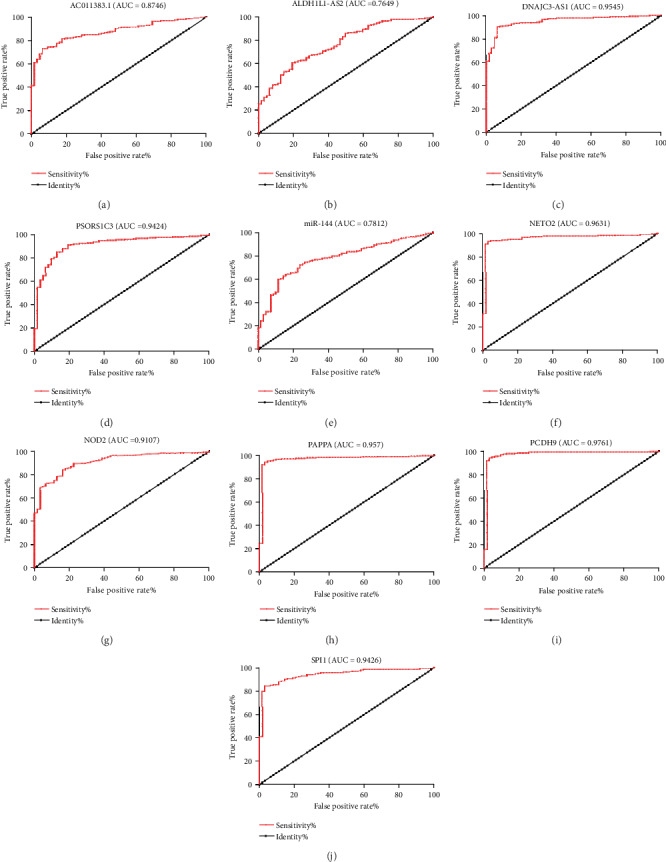
ROC analysis and AUC value of the ROC curve indicating the sensitivity and specificity for (a, b) DEmRNAs, (c, d) DElncRNAs, and (e) DEmiRNA in TCGA dataset. AUC: area under the receiver operating characteristic curves; ROC: receiver operating characteristic.

It is important to screen DEGs that could predict the prognosis of ccRCC. We used a univariate Cox proportional hazards regression model to analyze ccRCC patients' OS. There were 4 lncRNAs, 1 miRNA, and 5 mRNAs screened out: all of the lncRNAs, miR-144, and NETO2 positively influenced ccRCC patients' OS, whereas OS was negatively connected with NOD2, PAPPA, PCDH, and SPI2 (Figure 6). Compared with normal tissues, AC011383.1, PSORS1C3, miR-144, NETO2, NOD2, and SPI2 levels were increased in ccRCC tissues while ALDH1L1-AS2, DNAJC3-AS1, PAPPA, and PCDH9 levels were decreased (*P* < 0.05) (Figure 7). Receiver operating characteristic analysis was performed on these DEGs; their AUC value ranged from 0.736 to 0.976 (Figure 8, Table 5).

## 4. Discussion

The abnormal expression of protein-coding and protein-noncoding transcription is one feature of the cancer transcriptome [14]. The prognosis of ccRCC has been a big challenge due to the difficultly of diagnosis. So, it is significant to find effective biomarkers of ccRCC. In these years, dysregulated mRNAs [15], miRNAs [16], and lncRNAs [17] have been reported in ccRCC. There have been many reports of these RNAs on kidney tumor alone, but the interaction among mRNA, miRNA, and lncRNA is still unclear. Many of the genes with known ceRNA interactors have been found to be associated with liver cancer, leukaemias, lymphomas, and so on [18]. It is necessary to study the ceRNA network in ccRCC systematically.

In this study, 4 prognostic DElncRNAs (AC011383.1, ALDH1L1-AS2, DNAJC3-AS1, and PSORS1C3) were included in the ceRNA network. They may be independent prognostic factors of ccRCC patients' OS. Liang et al. reported that DNAJC3-AS1 plays a positive role in osteosarcoma evolvement by regulating DNAJC3. Moreover, it is a possible marker and treatment point for osteosarcoma [19].

Besides, we identified one independent prognostic DEmiRNA (miR-144). The upregulated miR-144 could enhance ccRCC malignancy and resistance to sunitinib by regulating ARID1A [20]. Nevertheless, Liu et al. believed that miR-144 inhibited cancer cell proliferation and metastasis in renal cell carcinoma [21]. The results in this study show that miR-144 has a positive impaction on ccRCC patients' OS.

We found 5 mRNAs that are connected with ccRCC patients' prognosis: NETO2, NOD2, PAPPA, PCDH9, and SPI1. Oparina et al. report that NETO2's expression level was increased in 90% ccRCC and 50% of papillary renal cancers. It is a possible biomarker in kidney cancer [22]. Mey et al. found that NOD2's expression level is higher in tumor tissues compared to normal tissues [23]. Dalgin et al. use microarray gene expression profiling to identify specific renal cell carcinoma markers. They identified 158 genes that dysregulated in tumor tissues; these genes are related to proteolysis and cell adhesion, including PAPPA [24].

However, there is no research about PCDH9 and SPI1 in ccRCC. In the present work, we found the two novel mRNAs as key predictors of ccRCC prognosis. PCDH9 is a member of the protocadherin family. It is associated with a kind of tumors, for example, melanoma [25], ovarian cancer [26], and medulloblastoma [27]. Ren et al. proved that PCDH9 is a tumor-inhibiting gene and has prognostic value in prostate cancer [28]. SPI1 is a member of the ETS family. It is relevant to multiple malignancies, for example, papillary thyroid carcinoma ^30^, pediatric T cell acute lymphoblastic leukemia [29], and breast cancer [30]. SPI1 is closely related to clinical features such as grade, metastasis, and stage, which means that SPI1 may be a potential prognostic biomarker of ccRCC.

A PPI network was built to illustrate the relationship between the mRNAs involved in the ceRNA network. Some of these mRNAs are closely connected with each other, suggesting that their interactions may play an important role in the development of ccRCC, which further confirms the significant role of this ceRNA network in ccRCC.

However, this study has some limitations. First, although we established the ceRNA network, we have not demonstrated other regulatory models. Besides, other studies may draw a different conclusion due to the differences in inclusion and exclusion criteria. Finally, there are few experimental data explaining the mechanisms of ceRNA, and we need further experiments to illustrate the role of ceRNA in ccRCC.

## 5. Conclusion

This article illustrated that lncRNAs, miRNAs, and mRNAs involved in the ceRNA network may be possible biomarkers. They are expected to predict the survival rate in ccRCC patients. Nevertheless, we need more experiments to validate these RNAs' biological function.

## Figures and Tables

**Table 1:** Top 10 upregulated and downregulated DEmiRNAs in ccRCC.

miRNA	Log_**2**_ fold change	*P* value	FDR	Regulation
hsa-mir-122	6.375971	4.90*E* − 79	1.80*E* − 77	Up
hsa-mir-875	4.236304	1.30*E* − 14	4.78*E* − 14	Up
hsa-mir-891a	4.144053	1.69*E* − 07	3.79*E* − 07	Up
hsa-mir-1293	3.96371	9.21*E* − 18	3.98*E* − 17	Up
hsa-mir-4773-2	3.781229	6.51*E* − 27	4.46*E* − 26	Up
hsa-mir-4773-1	3.717541	3.22*E* − 27	2.23*E* − 26	Up
hsa-mir-885	3.687125	2.37*E* − 36	2.42*E* − 35	Up
hsa-mir-891b	3.636965	2.34*E* − 05	4.25*E* − 05	Up
hsa-mir-599	3.590064	1.32*E* − 11	3.92*E* − 11	Up
hsa-mir-155	3.563706	2.20*E* − 67	4.79*E* − 66	Up
hsa-mir-514b	-6.01247	2.85*E* − 151	2.28*E* − 149	Down
hsa-mir-934	-5.74474	1.84*E* − 136	1.26*E* − 134	Down
hsa-mir-506	-5.66184	6.75*E* − 171	1.62*E* − 168	Down
hsa-mir-514a-3	-4.44858	3.71*E* − 162	4.44*E* − 160	Down
hsa-mir-514a-1	-4.42327	2.21*E* − 163	3.53*E* − 161	Down
hsa-mir-508	-4.41308	1.66*E* − 194	7.97*E* − 192	Down
hsa-mir-514a-2	-4.39209	1.57*E* − 152	1.50*E* − 150	Down
hsa-mir-129-1	-3.81098	7.60*E* − 77	2.27*E* − 75	Down
hsa-mir-129-2	-3.58243	6.41*E* − 66	1.33*E* − 64	Down
hsa-mir-509-3	-3.34887	1.86*E* − 116	1.11*E* − 114	Down

**Table 2:** Top 10 upregulated and downregulated DEmRNAs in ccRCC.

mRNA	Log_**2**_ fold change	*P* value	FDR	Regulation
GSG1L2	9.609768	7.03*E* − 23	4.67*E* − 22	Up
PAEP	8.674918	8.78*E* − 20	4.75*E* − 19	Up
CFHR5	8.510239	1.31*E* − 11	3.93*E* − 11	Up
HP	6.917233	6.19*E* − 19	3.17*E* − 18	Up
APOA4	6.896697	2.82*E* − 09	7.07*E* − 09	Up
RTL1	6.881453	1.18*E* − 12	3.83*E* − 12	Up
CRP	6.872065	2.01*E* − 20	1.13*E* − 19	Up
HHATL	6.715614	2.96*E* − 15	1.15*E* − 14	Up
ITIH1	6.707717	4.37*E* − 22	2.76*E* − 21	Up
CYP2A6	6.391803	5.14*E* − 20	2.83*E* − 19	Up
AQP2	-9.11281	1.07*E* − 112	1.96*E* − 110	Down
UMOD	-8.6196	4.97*E* − 101	6.96*E* − 99	Down
TMEM207	-8.47875	6.30*E* − 112	1.11*E* − 109	Down
ELF5	-7.71615	2.82*E* − 203	3.40*E* − 200	Down
MUC15	-7.38378	1.29*E* − 106	1.96*E* − 104	Down
ATP12A	-7.32714	3.14*E* − 178	2.18*E* − 175	Down
SEMG2	-7.27744	5.40*E* − 115	1.02*E* − 112	Down
NPHS2	-7.16242	1.65*E* − 77	1.25*E* − 75	Down
SOST	-7.10563	6.66*E* − 165	3.89*E* − 162	Down
PRR35	-6.98296	7.70*E* − 110	1.28*E* − 107	Down

**Table 3:** Top 10 upregulated and downregulated lncRNAs in ccRCC.

lncRNA	Log_**2**_ fold change	*P* value	FDR	Regulation
OSTM1-AS1	8.51552	6.61*E* − 52	2.06*E* − 50	Up
TTC21B-AS1	8.267223	2.84*E* − 77	1.83*E* − 75	Up
AC113410.2	7.658759	1.86*E* − 10	5.23*E* − 10	Up
AC008060.4	7.197464	1.38*E* − 14	5.38*E* − 14	Up
AL590644.1	6.998613	1.16*E* − 50	3.38*E* − 49	Up
AC090568.2	6.505535	1.94*E* − 15	8.03*E* − 15	Up
AP005233.2	6.444536	1.50*E* − 36	2.27*E* − 35	Up
AC008060.1	6.317642	3.47*E* − 16	1.53*E* − 15	Up
AC099786.2	6.188475	6.23*E* − 22	4.09*E* − 21	Up
AC073115.2	6.154044	2.60*E* − 43	5.43*E* − 42	Up
AC079310.1	-8.32279	1.06*E* − 170	3.50*E* − 168	Down
LINC02121	-8.32078	3.09*E* − 103	3.63*E* − 101	Down
LINC02437	-8.2957	2.95*E* − 184	1.25*E* − 181	Down
AC073336.1	-7.98709	3.05*E* − 125	5.15*E* − 123	Down
AC124017.1	-7.98205	7.52*E* − 256	9.60*E* − 253	Down
AC090709.1	-7.76666	1.53*E* − 260	2.27*E* − 257	Down
AC092078.2	-7.29943	7.89*E* − 204	4.15*E* − 201	Down
AC016526.1	-7.26561	6.32*E* − 279	1.41*E* − 275	Down
LINC01571	-7.03783	9.99*E* − 169	3.08*E* − 166	Down
LINC01543	-7.01222	1.10*E* − 152	2.72*E* − 150	Down

**Table 4:** KEGG pathway enrichment analysis of 33 DEmRNAs involved in the ceRNA network.

Term	ID	Input number	*P* value	Corrected *P* value
Pathways in cancer	hsa05200	5	0.0000	0.0009
Rheumatoid arthritis	hsa05323	3	0.0000	0.0009
EB virus infection	hsa05169	3	0.0002	0.0040
Proteoglycans in cancer	hsa05205	3	0.0002	0.0040
Bladder cancer	hsa05219	2	0.0003	0.0040
Human cytomegalovirus infection	hsa05163	3	0.0003	0.0040
Ras signaling pathway	hsa04014	3	0.0004	0.0040
Cytokine-cytokine receptor interaction	hsa04060	3	0.0007	0.0057
MAPK signaling pathway	hsa04010	3	0.0007	0.0057
MicroRNAs in cancer	hsa05206	3	0.0007	0.0057

**Table 5:** AUC value of DEmRNAs and DElncRNAs in TCGA dataset.

DEGs	SPI1	NOD2	NETO2	PAPPA	PCDH9
AUC	0.943	0.911	0.963	0.970	0.976
DEGs	miR-144	AC011383.1	PSORS1C3	ALDH1L1-AS2	DNAJC3-AS1
AUC	0.736	0.859	0.900	0.748	0.889

## Data Availability

Both RNA profiles data and clinical characteristics of ccRCC are publicly available and in open access platforms.
